# The Role of Scaffolds in Tendon Tissue Engineering

**DOI:** 10.3390/jfb11040078

**Published:** 2020-11-01

**Authors:** Angelo V. Vasiliadis, Konstantinos Katakalos

**Affiliations:** 1 School of Medicine, Aristotle University of Thessaloniki, 54124 Thessaloniki, Greece; 2 2nd Department of Orthopedic Surgery, General Hospital of Thessaloniki, 56403 Thessaloniki, Greece; 3 Laboratory for Strength of Materials and Structures, Department of Civil Engineering, Aristotle University of Thessaloniki, 54124 Thessaloniki, Greece; kkatakal@civil.auth.gr

**Keywords:** tendon injury, biomaterials, scaffolds, tissue engineering

## Abstract

Tendons are unique forms of connective tissue aiming to transmit the mechanical force of muscle contraction to the bones. Tendon injury may be due to direct trauma or might be secondary to overuse injury and age-related degeneration, leading to inflammation, weakening and subsequent rupture. Current traditional treatment strategies focus on pain relief, reduction of the inflammation and functional restoration. Tendon repair surgery can be performed in people with tendon injuries to restore the tendon’s function, with re-rupture being the main potential complication. Novel therapeutic approaches that address the underlying pathology of the disease is warranted. Scaffolds represent a promising solution to the challenges associated with tendon tissue engineering. The ideal scaffold for tendon tissue engineering needs to exhibit physiologically relevant mechanical properties and to facilitate functional graft integration by promoting the regeneration of the native tissue.

## 1. Introduction

Tendons exhibit superior mechanical strength and flexibility in order to perform their pivotal role as an active element in joint stability during movement and physical exercise [1]. Within the orthopaedic field, tendinopathy is one of the most frequently self reported musculoskeletal injuries in the athletic and working populations, which result in pain, swelling and impaired function [2]. More than 30 million tendon injuries are reported annually worldwide, while the actual number might be even higher since many injuries are not reported [3]. The elderly population and athletes are predominantly affected by tendon injuries. In fact, it is estimated that approximately 50% of all sports injuries involve tendons due to overuse [4]. It is believed that population ageing and the overall increase in sports activities, such as running, is contributory to this rising incidence [4,5].

Tendons are bands of dense fibrous connective tissue that are attached to every muscle through the myotendinous junction and to every bone through the enthesis, where their primary function is to resist tensile forces ensuring joint movement. The ability of tendons to sustain and transmit large tensile forces for long periods is also closely linked to damage by microtrauma and finally rupture [4,6]. Currently used therapies are mainly conservative and include analgesia, stretching exercise and activity modification [6]. Surgical repair is recommended in case the conservative treatment fails to adequately relieve symptoms and restore function [7]. However, surgery remains a questionable and imperfect solution due to reduced mechanical strength, possible wound infection and donor site morbidity [8].

In this regard, tissue engineering strategies, including the concept of scaffold induced endogenous tissue repair, have gained popularity due to their assured biocompatibility and bioactivity [1,8]. Scaffolds can be used as a suitable platform, which permits cell-biomaterial interactions, cell adhesion, proliferation, and differentiation, while provoking a minimal degree of immune response [1,9,10]. Preliminary studies have shown that scaffolds may be used as an alternative augmentation, associated with significant therapeutic potential [9]. The scaffolds can mimic the extracellular matrix of the surrounding environment in terms of composition and architecture. Furthermore, they pose cell adhesion sites, in order to promote cellular adhesion and proliferation [1,9]. Nevertheless, successful tendon repair (restoration of the functional, structural and biomechanical properties of tendon) that will lead to a clinically effective and commercially successful product in orthopaedics, remains a significant clinical challenge.

## 2. Basic Tendon Structure and Function

Tendons play a crucial role in the musculoskeletal system by transmitting muscle forces to the skeleton. There are approximately 4000 tendons and ligaments in the human body, but the exact number count depends on a person’s size and muscle mass [5]. Specifically, tendons consist of dense regular connective tissue, allowing the movement and maintenance of the body posture, as they transmit the forces produced by the muscular contraction to the skeletal bones [2].

The tendon consists of two major types of cells; tenocytes, terminally differentiated cells, which constitute the main cell type of the healthy mature tendon (90–95% of the cellular content) and require a mechanical stimulus for their proper function, as well as tendon stem/progenitor cells, which reside in the extracellular matrix of the tendon. Other cell types include synovial cells on the tendon surface, chondrocytes at the regions of pressure and insertion and vascular cells in the endo- and epitenon regions [11]. Under normal conditions, tenocytes are responsible for maintaining tendon homeostasis and repair, while tendon stem/progenitor cells play a vital role in tendon maintenance and repair by undergoing self-renewal and differentiation into tenocytes [11,12].

**Figure 1:**
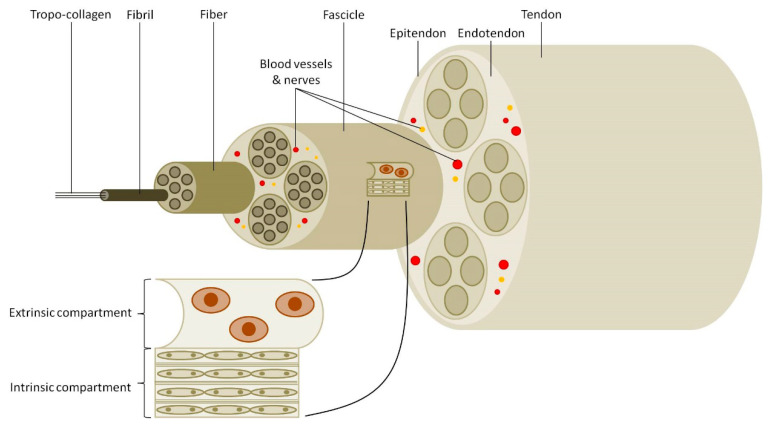
Tendon hierarchical structure. A simplified model of tendon structure showing collagen molecules to represent the simplest forming structure of tendon with complex arrangement up to tendon fascicles producing the final tendon tissue. Tendon fascicles represent the basic unit comprising the “intrinsic compartment” (tenocytes and collagen fibers run in parallel arrays). The “extrinsic compartment” consists of synovium-like tissues connecting the immune, vascular and nervous systems.

Healthy adult tendons are fibrous tissues with the structural hallmark of parallel packed arrays of collagen fibrils containing mostly type I collagen, 70% by weight, with type III and V collagens throughout and provide it with the unique combination of a tensile strength and flexibility [4,9]. A bunch of collagen fibrils forms a collagen fiber, while fibers form fascicles and bundles of fascicles form the fascicular matrix. The endotendon is a thin layer which contains lymphatic, blood vessels and nerves, which occupies the space between fascicles bundles, thus allowing them to make small slip motions. The endotendon tissue continues in the form of an epitendon, which is the glistening, synovial-like membrane that envelops the tendon surface and prevents adhesion to the neighboring tissues (Figure 1) [5]. Therefore, the tendon is a complex physiological system consisting of an “intrinsic compartment” (tenocytes and parallel collagen fibers) and an “extrinsic compartment” (synovial tissue connecting immune, vascular and nerve system), with a possible synergism between them in the maintenance of a healthy tissue (Figure 1) [2].

**Figure 2:**
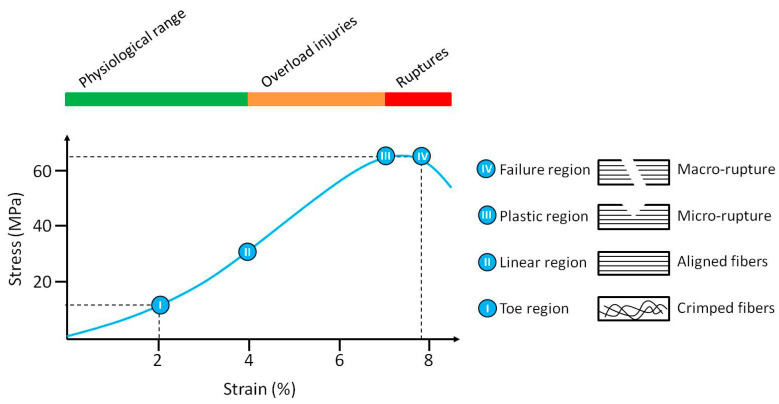
A typical stress-strain curve and a schematic representation of the behavior of the collagen fibers for tendon tissue. At strains of up to 2%, the collagen fibers the of tendon are crimped (toe region). When the load applied to the tendon increases (below 4%), the collagen fibers start to align with each other while losing the crimped behavior. In this region, the collagen fibers provide a quite ideal elastic recovery, if load is removed (linear region). At strains above 4%, the collagen fibers begin to experience destructive changes, e.g., micro-rupture in the collagen network. In this region, the changes are irreversible in the tissue (plastic region). If loading continues further, the tissue may permanently deform until the complete failure of the tendon, e.g., macro-rupture in the collagen fibers (failure region).

The structure and composition of the tendons are responsible for their unique mechanical properties, reflected by four distinct regions of the stress/strain curve. The first region is known as the toe region, where the tendon is strained up to 2%. This region represents the stretching-out of the crimp-pattern of the composing fibers. The second region is called linear region, where the tendon is stretched less than 4%. In this region, all fibers have been recruited and are straight. When the stress is further increased from the linear region, the slope of the curve changes and the plastic region begins. Stretching over 4% can result in microscopic tearing within the tissue leading to the development of tendinopathy. As the strain on the fibers continues, macroscopic failure occurs and eventually the tendon is ruptured (Figure 2) [2,4,13]. Mechanical signal transduction through molecular signaling triggers tendon adaptive responses in mechanical loads [12]. However, the overloading of a tendon, either by exceeding its maximum strain or by providing insufficient recovery time between repetitive sprains can cause damage to the collagen network, which is widely associated with a decrease in quality of life [1,2,12].

## 3. Tendon Healing: Repair and Regeneration

**Figure 3:**
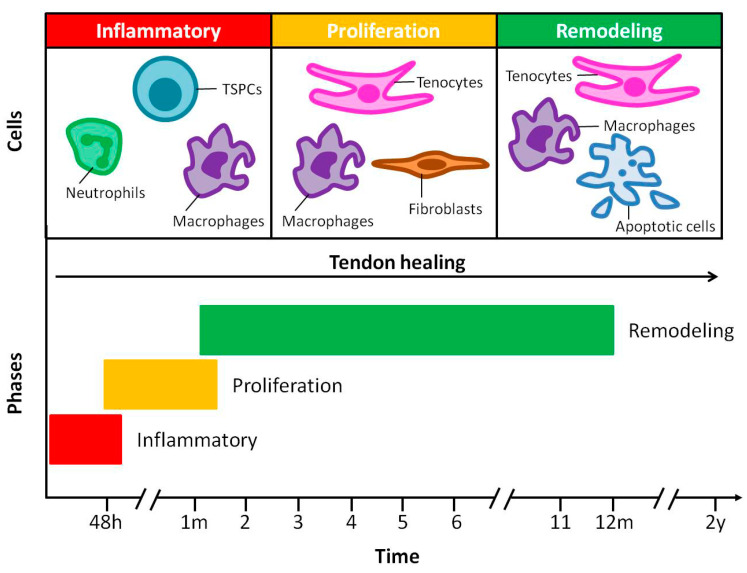
Overview of the tendon repair process in humans. The healing of ruptured tendons passes through three main overlapped phases containing distinctive cell and molecular cascades. Their duration depends upon the location and severity of the tendon injury.

The healing of a ruptured tendon after an injury or surgical repair usually includes three sequential but overlapping phases: (i) inflammation, (ii) proliferation and (iii) remodeling [4,11]. During the inflammation phase, which begins immediately after the injury, the hematoma formation is critical for the healing process. Various cells, including neutrophils, monocytes/macrophages and tendon stem/progenitor cells (TSPCs type I and II), are attached to the site of the injury by pro-inflammatory cytokines, including interleukin-6 and interleukin-18 [11,14]. After being released into the blood vessels, neutrophils contribute to tissue injury by amplifying the inflammatory response and removing foreign cells. At the baseline, macrophages remove from the tissue dead cells necrotic tissue and toxic metabolites via phagocytosis. However, after injury, these homeostatic functions are amplified by a variety of mediators in order to facilitate tissue repair. During the inflammatory phase, tendon stem/progenitor cells might undergo differentiation to tendon-like cells and play an essential role in tendon maintenance, regeneration and repair. This phase is followed by the phase of proliferation. During this phase, macrophages and endothelial cells release growth factors, in order to direct cell recruitment and form granulation tissue within the injured region, to serve as a provisional matrix during the healing process. Thereafter, fibroblasts and tenocytes are recruited to the injured region to produce collagen type III, fibronectin and proteoglycans in order to initially create an unorganized extra-cellular matrix and bridge the injured region. After that, the collagen type III is replaced by the stronger collagen type I. Finally, the remodeling phase, which is marked by the alignment of tenocytes and collagen fibers, an increase in the proportion of collagen type I and a decrease in cellularity and collagen type III, can continue for years. Furthermore, macrophages have been found to play a key role in tissue development and homeostasis by phagocytosing necrotic and apoptotic cells and to participate in the remodeling of the tissue (Figure 3) [11,14,15].

Despite the natural healing progress or an optimal treatment after surgical repair, the collagen fibers in the tendon may be deficient in content, quality and alignment [1,16]. As a result, the structural and biomechanical properties of the healed tendon might be inferior to those of the native tendon. Considering that the healed tendon is often characterized by increased risk of tendon degeneration, late complications include functional impairment and the risk of re-rupture at the site of injury or near the region of injury during later activities [1,2,4,16].

## 4. Requirements of Scaffold for Tendon Repair

**Figure 4:**
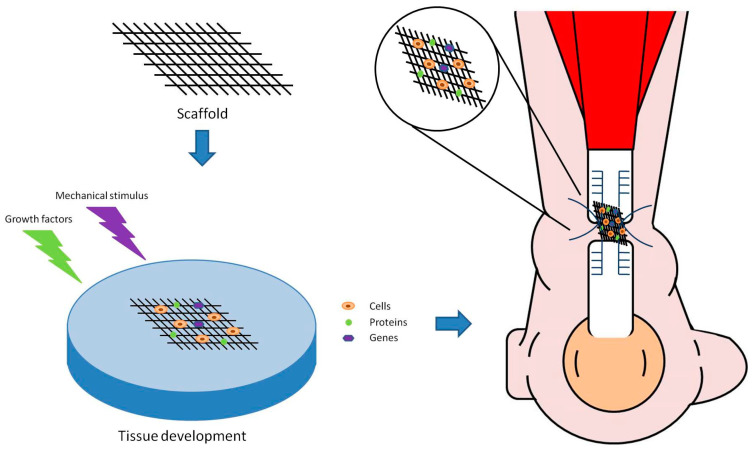
An Achilles tendon rupture. Tissue engineering strategy for tendon regeneration, which includes implants and contains a combination of cells, proteins and scaffold materials, which can be directly implanted and sutured in the side of the ruptured tendon.

Numerous treatment approaches have been proposed to improve tendon healing, including surgical techniques, growth factor- and cell-based therapies, biological- and synthetic-based scaffolds and rehabilitation protocols [1,9,17,18]. However, data on these approaches are controversial and the optimal treatment for a successful restoration of the joint anatomy has yet to be defined. Tissue engineering using 3D structured scaffolds represents a promising strategy for achieving biological fixation and integrative soft-tissue repair in people who sustained a tendon injury [1,9]. Tissue engineering exploits the production of ex vivo functioning artificial tissues, such as bio-responsible scaffolds. These can then be implanted at the site of injury, to enable the in situ restoration and improve the function of de novo tissue (Figure 4) [4,9].

First of all, any scaffold used for tissue engineering needs to induce a positive biological response and accelerate the healing process after implantation. For the above reason, the implanted scaffold must have sufficient mechanical integrity and exhibit excellent biocompatibility and biodegradability [1,9,19]. After implantation, the ideal scaffold must elicit a negligible immune response, in order to avoid severe inflammatory responses that might reduce healing properties and/or cause material rejection [8,19]. Beyond their safety, scaffolds should provide appropriate structural support, and in certain cases, biomechanical cues, to promote the safe and effective reconstruction of a functional tissue in vivo [9,10,20]. A way to promote scaffold integration is to modify the chemical backbone of the biomaterial or the physicochemical properties of the surface to influence cell adhesion, migration, proliferation and differentiation in vivo [21]. Ideally, scaffolds should be able to withstand physiological loads and to integrate with the adjacent host tissues following in vivo implantation, without disrupting the biological repair [8].

## 5. Different Designs of Scaffold and Current Progress

According to the type of the biomaterial used, scaffolds can be broadly divided into three groups: (i) biological, (ii) synthetic and (iii) composite. Designing a scaffold should meet several requirements for tendon tissue engineering and is still largely open [1,19]. Scaffolding biomaterials must be biocompatible and promote rapid and effective integration of the new tissue without carrying a risk of inducing an immune response [19,22]. Another critical aspect is the need for an appropriate void space within the scaffold structure to allow nutrient delivery and promote uniform cell delivery and tissue ingrowth [19,23].

Furthermore, biodegradability is an important feature of scaffold fabrication, which falls in line with adequate mechanical properties of the scaffold. Following implantation, the scaffold must degrade over the time at a controlled resorption rate and be replaced by newly regenerated tissue [1,20,24]. Hence, the ideal goal of tissue regeneration with the use of scaffolds is to develop the optimal characteristics (e.g., strength, elasticity, density) and mimic the structure of the previous healthy tissue [25].

### 5.1. Biological Scaffolds

Biological scaffolds, derived from mammalian tissues, such as human, porcine, bovine and equine, have been successfully used in both pre-clinical animal studies and human clinical trials [9,19,26]. In order to minimize the risk of host rejection while maintaining their complex collagenous architectures and mechanical properties, tissues such as pericardium and intestine are processed through a cascade, including general cleaning, removal of cell components, fat, lipids as well as endotoxins [26]. Arthroflex^®^ (human, dermis), Dermaspan™ (human, dermis), BioArthro™ (human, amniotic membrane), TissueMend^®^ (bovine, fetal dermis) or Restore™ (porcine, small intestinal submucosa) are biological scaffolds that are approved by the FDA for tendon application [27]. Mechanical properties are the major limitation of these biological scaffolds, especially compared to those of normal tendons (Table 1) [1,7,28].

Naturally occurring scaffolds, such as accellular dermal matrix (GraftJacket™, Wright Medical Technology, Inc., Memphis, TN, USA), have been clinically used as augmentation devices in chronic Achilles tendon ruptures leading to early return to activities and improved plantar flexion strength [26,29]. Once more, the promising results reported from human dermal matrix scaffolds have been supported by the absence of adverse inflammatory or septic reactions [30]. Several studies also evaluated the role of biological scaffolds in large or massive rotator cuff tears. These studies showed that scaffolds not only reinforce the mechanically defective part of the tendon but also stimulate its intrinsic healing potential [26,31].

A study by Metcalf et al. on 12 patients using a pleuripotent xenograft (porcine small-intense submucosa) did not show any postoperative adverse events [32]. However, in this study, the patients underwent repair of their massive chronic rotator cuff tear. Magnetic resonance imaging (MRI) in the two year follow-up has shown tendon healing with incorporation of graft in 11 patients. Only one patient showed complete resorption of the graft within 12 weeks from surgery. The mean post-operative University of California, Los Angeles (UCLA) score was increased from 9.3 to 19.9 on a scale of 1 to 35 and was statistically significant (*p* = 0.01) [32]. Gupta et al treated 26 patients with massive or full thickness rotator cuff tears and minimal glunohumeral arthritis with dermal tissue matrix xenografts [33]. With a mean follow-up of 32 months, sixteen patients (73%) demonstrated fully intact tendon graft reconstruction, while five patients had partially intact tendon graft reconstruction. Only one patient had a complete tear at the graft-bone interface. Using a human dermal allograft interposition repair of massive rotator cuff tears, the mean pain level was decreased (*p* = 0.002), the mean range of motion was improved (*p* = 0.001) and the mean supraspinatus and external rotation strength improved (*p* = 0.001). The mean American Shoulder and Elbow Score (ASES) and Short Form-12 (SF-12) score were also improved (*p* = 0.0003 and *p* = 0.03, respectively) [33].

Lee et al. used an acellular human dermal tissue as an augmentation material in neglected Achilles tendon repair in nine patients and followed-up for a minimum of 20 months [29]. There were no reported cases of re-rupture or recurrent pain. The average return to activity time was approximately 15 weeks. The study by Brigido et al. also described an augmentation reconstruction technique of the Achilles tendon for chronic Achilles tendinosis using a human dermal graft [34]. There were 21 patients with good clinical and patient-reported short-term (24 weeks) results who returned to full activities in 12 weeks. In another case series of nine patients who underwent Achilles tendon repair with accellular dermal matrix augmentation, no re-ruptures or complications were reported in any of the patients with a minimum follow-up period of 2 years [35].

The decellularized allograft tissues are considered to be a type of biological scaffold used for tendon regeneration with the advantages of similar mechanical properties [1,9,36]. In addition, the endogenous integrin binding sites are present in the native extra-cellular matrix. The use of native extra-cellular matrix as a scaffold for tissue engineering and regenerative medicine presents an attractive material option due to their ability to provide structural support and regulate cell viability and metabolism [1,9,37]. As a new biological scaffold, the extra-cellular matrix has been shown to promote the attachment, migration and proliferation of progenitor cells when implanted in the site of the injury. The release of signaling molecules that modulate the innate immune response and recruit progenitor cells to the site of scaffold remodeling has been implicated as a possible trigger mechanism for this process [38,39].

### 5.2. Synthetic Scaffolds

Unlike biological scaffolds, synthetic scaffolds in graft-augmentation appear to have stronger mechanical properties (Table 1). Despite this advantage, their limited biocompatibility and degradation products can lead to an immunological response interaction with the surrounding tissues, including host tissue integration [1,9]. Tissue engineering has contributed alternative strategies for tendon repair in order to overcome this issue. Synthetic scaffolds are typically more versatile because of their chemical and physical properties which can be tailored with a high degree of precision [1,9,19].

Many synthetic polymers have been handled in order to build functional bioengineered scaffolds for tendon repair strategies, including polystyrene, poly-l-lactic acid (PLLA) [40], polyglycolic acid (PGA) [41] and poly-dl-lactic-co-glycolic acid (PLGA) [42]. PGA is one of the most widely used scaffolding polymers. However due to its relatively hydrophilic nature [41], other degradable polymers, including PLLA and PLGA, have emerged as more promising candidates for tendon engineering [9]. In order to exert more control over the mechanical properties of these types of scaffolds, two main approaches are applied. In the first one, the structure of the material is optimized through scaffolds to improve efficacy and safety, while in the second approach, the chemistry is reformed [22].

We must also consider the differences in the mechanical properties between different polymers. Promising results using scaffolds with tendon-like properties in order to improve rotator cuff healing were reported using mathematical modeling [43]. Scaffold incorporation with reduced or supraphysiologic mechanical properties (i.e., stiffness, strength, yield load) did not translate into stiffer or/and stronger [1,43]. It is important that the scaffold exhibits appropriate mechanical properties. The degradation kinetics of scaffolds must also be carefully considered in scaffold final design, since many synthetic scaffolds degrade to cytotoxic components, which limit their in vivo applicability [9]. Recent studies demonstrated that a PGA scaffold demonstrated a higher number of cells attached to its surface. By contrast, scaffolds made from PLA demonstrated slower degradation rates. PLGA is a co-polymer of PLA and PGA with the advantage of achieving desirable properties and functionalities, such as degradation rate, hydrophilicity and mechanical strength, through modification of PLA:PGA ratio [1,19,41]. Therefore, an ideal polymer provides the optimum combination of degradation rate and speed of tissue ingrowth.

A recent study by Proctor showed promising results at a follow-up of 42 months after arthroscopic repair of large and massive rotator cuff tears augmented by the use of a PLLA synthetic scaffold [44]. A combination of ultrasound and MRI showed that fourteen patients (78%) had an intact rotator cuff at 42 months after surgery, while functional outcome, assessed by the American Shoulder and Elbow Surgeons (ASES), was a shoulder score of 82 out of 100 points. In a preliminary study, the aforementioned scaffold was evaluated in sixteen patients with massive or recurrent rotator cuff tear who underwent open repair with synthetic PLLA scaffold [45]. The mean ASES and Penn shoulder Score were significantly improved (*p* = 0.0001 and *p* < 0.005, respectively) with an average follow-up of 1.5 years, despite a re-rupture rate of 62%.

Despite the advantages that degradable polyesters offer, several limitations need to be overcome. First, their hydrolysis releases acidic degradation products, which, in high concentrations, have been found to be toxic to both tenocytes and osteoblasts [9,19]. Second, a common issue with synthetic scaffolds is their poor physiological activities, such as the selective cell adhesion (e.g., collagen and fibrin) [1,9]. To overcome these limitations, alternative scaffold sources and fabrication methods have been investigated.

### 5.3. Composite Scaffolds

Apart from biological and synthetic scaffolds, composite scaffolds, resulting from the composition of two or more materials, are widely used in tissue engineering. Composite scaffolds mimic in vivo micro-architecture of the native tissue and influence cellular function, mechanical properties of the tissue and the integration of grafted engineered tissue with the host more closely than any other available material [37]. It is important to note that composite scaffolds are designed with a specific purpose, to control degradation rates, appropriate mechanical properties, ensure non-immunogenicity and saturability in tendon tissue engineering [8].

Recently, many scientific studies show a trend in the development of scaffolds made of polymer-ceramic composite materials for tissue engineering [24,46]. This is because ceramics have several outstanding merits, such as osteoinductive properties, excellent biocompatibility, resistance to corrosion and high compression. However, their use is limited by their low degradability and poor mechanical strength that adversely influences tissue stabilities [28,46]. However, polymers pose good mechanical properties, e.g., stability, and exhibit a much slower degradation rate, but poor osteointegration [24,28,46]. Thus, the development of ceramic-polymer composites as scaffold materials for tissue engineering, enable the use of biodegradable materials with valuable mechanical and biological properties.

## 6. Conclusions

Tendon injuries represent the most frequent musculoskeletal condition for which patients seek medical attention. These injuries also present significant healthcare challenges for clinicians due to potentially slow healing rates, loss of function and scar tissue formation around the site of trauma. Biomaterials, such as scaffolds, represent a major component of tendon tissue engineering. The accurate selection of the appropriate type of scaffold by the clinician will be based on its relative advantages and limitations. The ideal scaffold should mimic the properties of the native tissue, not only in terms of mechanical function, but also proper topography, geometry and porosity to recreate the native microenvironment and aid the cell adhesion, growth and differentiation of the populating cells. Therefore, future research should be focused on determining the optimum combination of regenerative factors (cells, growth factors, genes) and the ideal scaffold to orchestrate the complex chain of tendon tissue engineering, ultimately leading to significant improvement in clinical outcomes. 

## Figures and Tables

**Table 1:** Advantages and disadvantages of biological versus synthetic scaffolds.

Type of Scaffold	Advantages	Disadvantages
Biological	High hydrophilic properties Low immunological response Cell adhesive	Low mechanical properties
Synthetic	High mechanical properties Versatility	Low hydrophilic properties High immunological response Selective cell adhesive
